# Association of Tarlov cyst with cauda equina syndrome and spinal cord infarction following caudal epidural block: A case report

**DOI:** 10.1097/MD.0000000000035824

**Published:** 2023-11-17

**Authors:** Sunyoung Joo, Chung Reen Kim, Sunyoung Kim

**Affiliations:** a Department of Rehabilitation Medicine, Ulsan University Hospital, University of Ulsan College of Medicine, Jeonha 1(IL)-dong, Dong-gu, Ulsan, Korea; b Department of Neurology, Ulsan University Hospital, University of Ulsan College of Medicine, Jeonha 1(IL)-dong, Dong-gu, Ulsan, Korea.

**Keywords:** case report, cauda equina syndrome, epidural injection, infarction, Tarlov cysts

## Abstract

**Rationale:**

Caudal epidural block (CEB), which injects drugs into the epidural space through a sacral hiatus, is considered a safer alternative to other approaches. Serious complications, such as cauda equina syndrome or spinal cord infarction, have been reported very rarely, but their coexistence after CEB, which may be related to the ruptured perineural cyst, also known as a Tarlov cyst, was not reported.

**Patient concerns:**

A 40-year-old male patient presented with bilateral lower extremity radicular pain. CEB was performed without image guidance. The patient exhibited sensory deficits below L2, no motor function (0-grade), hypotonic deep tendon reflexes, and no pathological reflexes.

**Diagnoses:**

Spinal cord infarction, cauda equina syndrome, and sacral level perineural cyst with hemorrhage.

**Intervention:**

High doses of steroids and rehabilitation were performed.

**Outcomes:**

The patient was discharged after 28 days with persistent bilateral leg paralysis and sensory deficits below the L2 level. The patient demonstrated no neurological improvement.

**Lessons:**

Magnetic resonance imaging, including the sacral area, should be performed before performing CEB, to confirm the presence of a perineural cyst.

## 1. Introduction

Lumbar radicular pain is commonly caused by spinal nerve root irritation due to intervertebral disc pathology, followed by lumbar spinal stenosis.^[1]^ Further, epidural steroid injection (ESI) is one of the most common nonsurgical treatment methods that manage radicular pain by injecting steroids or anesthetics into the epidural space surrounding the spinal nerve.^[2]^ Mainly translaminar and transforaminal approaches are used under fluoroscopic guidance and are generally effective and safe. However, various severe complications were reported, such as dura puncture, stroke, spinal cord infarction (SCI), and nerve root damage.^[3]^ Thus, the caudal epidural block (CEB) is often considered a safer alternative for those approaches.^[4]^ CEB is another ESI technique in which a needle is inserted through the sacral hiatus and steroids are injected into the epidural space. Drugs could be successfully injected into the epidural space without deeply inserting a needle because the caudal sac usually ends at the S2 level.^[5]^ Thus, CEB is considered a safe procedure even when blindly performed.^[6]^ Reported severe complications are few compared to translaminar and transforaminal approaches,^[4,7]^ but several cases of cauda equina syndrome (CES) following CEB have been reported.^[8,9]^ Additionally, a case of SCI after a CEB was reported, although very rare, in a patient with tethered cord syndrome.^[10]^ Recently, we experienced a patient with paraplegia immediately after a CEB. We considered the paraplegia to be caused by SCI and CES. Additionally, the occurrence of hemorrhage in the sacral Tarlov cyst may be related to this case, and this association has never been reported. Thus, we would like to report this unusual case for further discussion. This case report was approved by the institutional review board with a waiver for informed consent (IRB no. 2023-06-043).

## 2. Case report

**Figure 1.**
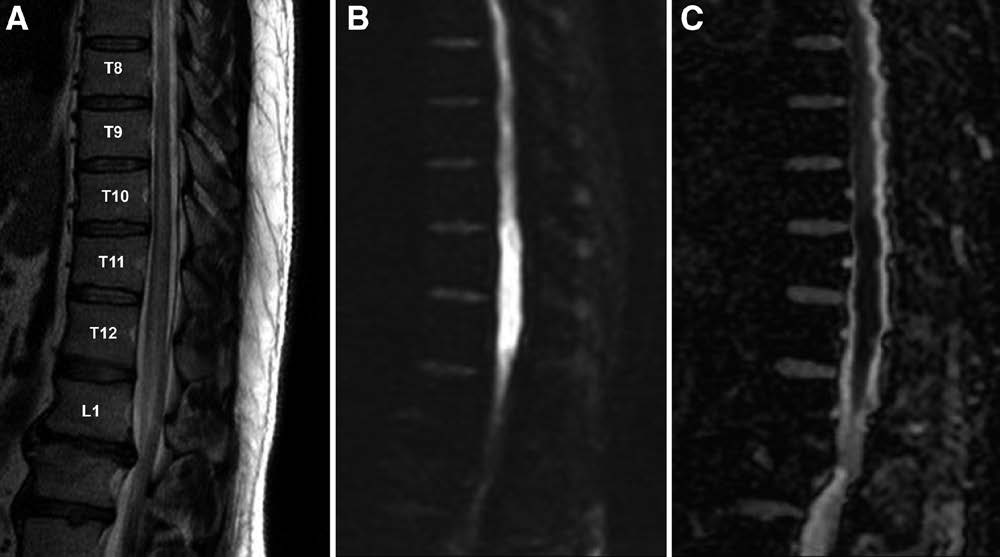
Sagittal magnetic resonance images. T2-weighted sequences show high signal intensity lesions within the central gray matter of the thoracic cord, extending from the T9 to L2 levels. (A) And high signal intensity on the sagittal diffusion-weighted image and low signal intensity on the apparent diffusion coefficient map demonstrates the acute phase of spinal cord infarction. (B and C).

**Figure 2.**
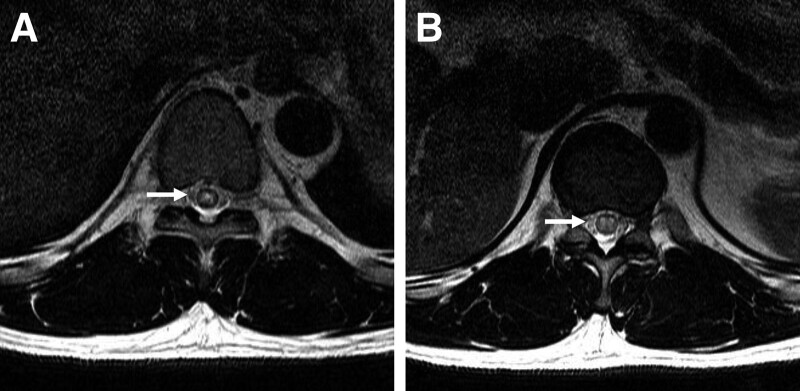
Axial magnetic resonance images at T9 (A) and T11 (B) levels. T2-weighted sequences show the classic “owl’s eye” pattern within the gray matter.

**Figure 3.**
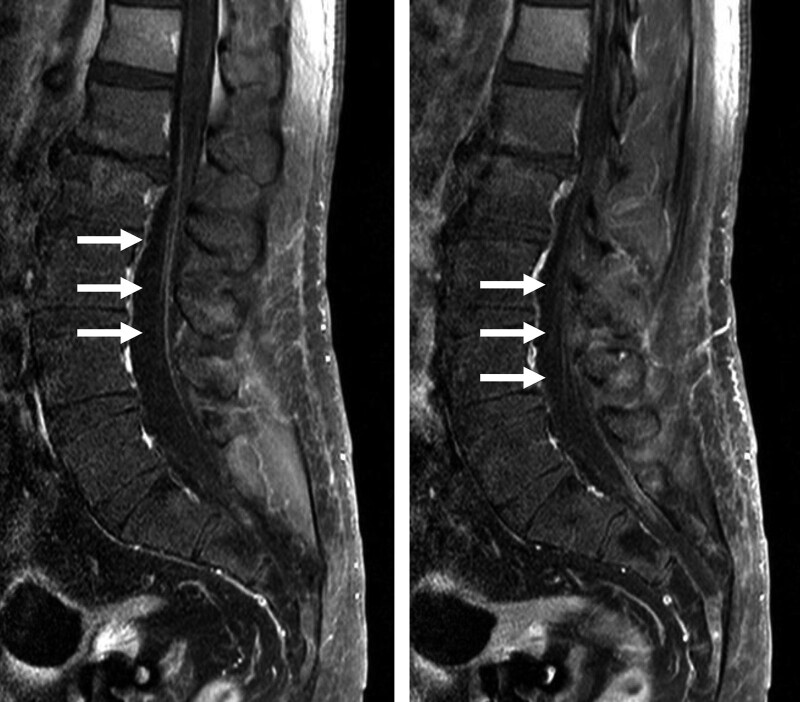
Enhancement of cauda equinae is observed on T1-weighted contrast-enhanced images.

**Figure 4.**
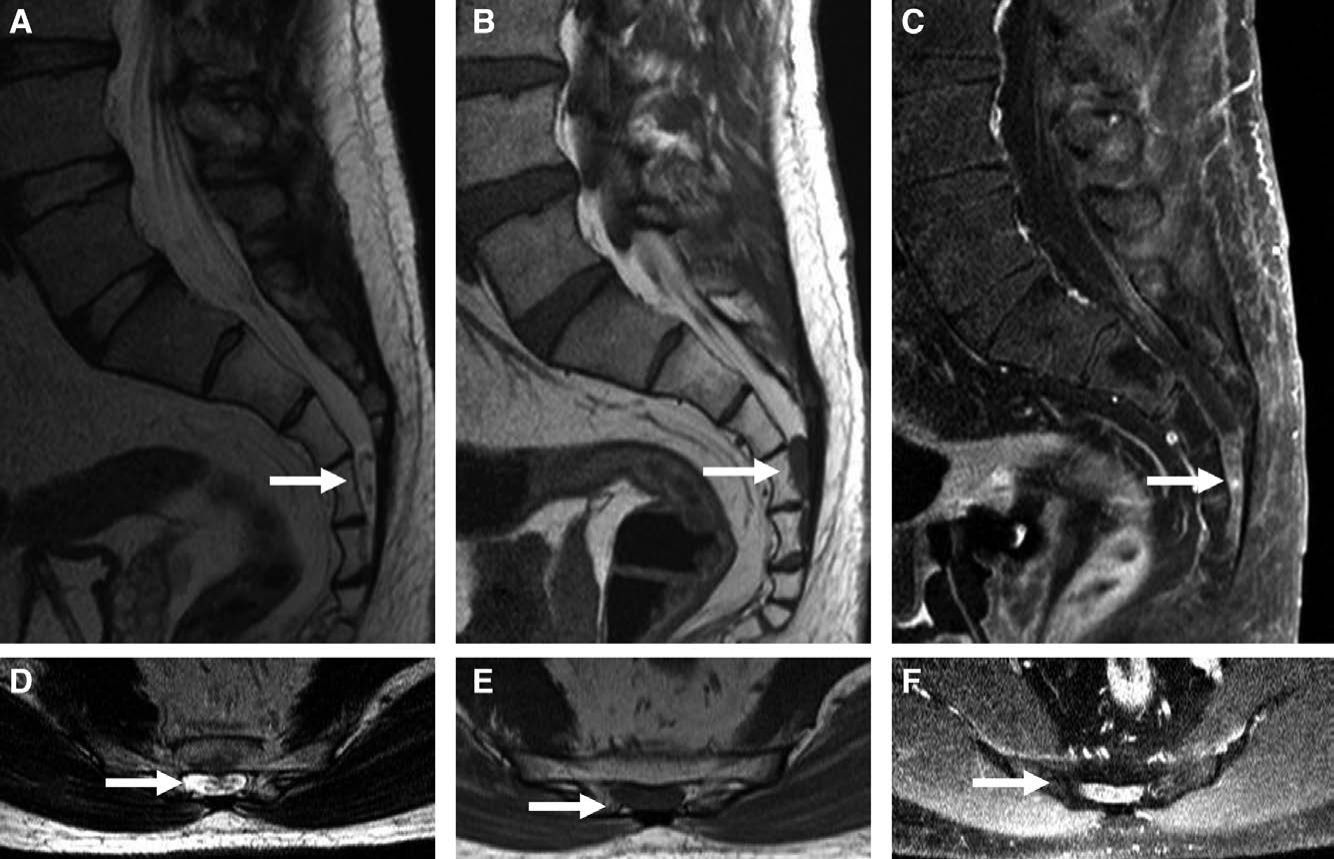
Sagittal and Axial T2-weighted, T1-weighted, and T1-weighted contrast-enhanced images. Hyperintense T2-weighted and isointense T1-weighted images show a perineural cyst with acute hemorrhage that originated from the perineurium at the S4–S5 levels (A, B, D, and E).

A 40-year-old male patient was admitted to our hospital due to weakness and sensory loss of both lower extremities after CEB. He had suffered chronic back pain and has got intermittent ESI for pain control over the past several years. However, he had experienced no significant side effects from ESI. He underwent another CEB at a local orthopedic clinic to manage his back pain just before visiting our hospital. The caudal ESI was performed blindly with a total volume of 16 mL, which included lidocaine 2% at 1.3 mL, dexamethasone at 0.5 mg, and a small amount of triamcinolone (approximately < 40 mg) mixed with normal saline. The patient experienced a tingling sensation throughout his body immediately after the injection, and bilateral leg paralysis developed subsequently. He felt no sensation below the L2 level and had zero motor grade on both lower extremities upon admission. Additionally, deep tendon reflexes and pathological reflexes were absent at both lower extremities. He also failed to urinate, so a Foley catheter was inserted. Initial T2-weighted images of the whole spinal magnetic resonance images (MRI) revealed a high signal intensity in the spinal cord from T9 to L2 levels, indicating SCI (Figs. 1 and 2). Additionally, a cauda equina enhancement was revealed from L1 to S2 levels (Fig. 3), as well as an perineural cyst (Tarlov cyst) with hemorrhage measuring 2.8 cm in length in the spinal canal at the S4 to S5 levels (Fig. 4). He had no other significant past medical history or medications, as well as any specific family history. The cerebrospinal fluid examination revealed a white blood cell count of 5/μL, glucose level of 66 mg/dL, chloride concentration of 125.5 mEq/L, and a slightly elevated T protein concentration of 338.9 mg/dL, without an increase in white blood cells. The venous blood test revealed no specific findings except for an increased proportion of polysegmented neutrophils (92.2%), with a white blood cell count of 5730/uL.

Oral methylprednisolone at 100 mg daily was administered for 4 days and tapered for 10 days to manage the cauda equina inflammation. The follow-up MRI conducted 1 day after admission showed an increased area of high signal intensity in the spinal cord from T7 to L2, along with reduced enhancement of the cauda equinae. One week after admission, the patient was transferred to the Department of Rehabilitation Medicine and underwent physiotherapy and occupational therapy after initial neurologic workup and management. Functional improvement was observed 4 weeks after onset, and he could transfer from bed to wheelchair with minimal assistance. However, motor power, sensory function, and bowel and bladder functions were not improved.

## 3. Discussion

CEB is generally a safer procedure for treating lumbar stenosis and offers an alternative to lumbar transforaminal or translaminar techniques.^[8]^ However, several cases of uncommon complications after CEB have been reported, and one of them is a CES.^[8,9,11]^ CES is a neuropathy caused by the compression of multilevel cauda equina filaments below the L1 level, resulting in various symptoms, including lumbar back pain, radiating pain to the lower extremities, motor impairment, paresthesia, and difficulty in voiding and defecation.^[8]^ CES mainly occurs immediately after the injection of a drug mixture or within a few days after the procedure.^[8]^ The development of CES after CEB can be explained by meningitis, osmotic pressure, direct toxicity of injected drugs, injury due to intradural or intraneural injection, or hematoma.^[12]^ In this case, hemorrhagic rupture of the perineural cyst might occur due to needle insertion, and the heme in the blood clots may have caused inflammation and neural damage of the cauda equina.^[13]^ Cauda equina enhancement on MRI, along with flaccid muscle tone and lack of deep tendon reflexes in both lower extremities, are thought to support the presence of CES in this case.

However, CES is not the only problem for the patient. T7 to L2 SCI, which is an even rarer occurrence than CES, was also observed after CEB.^[7]^ Well-known probable mechanisms of SCI are needle-induced vasospasm, particulate steroid embolization, mechanical disruption of radiculomedullary arteries, and compression from an epidural abscess or hematoma.^[14]^ Among them, one of the most common causes is particulate steroid embolization. Particulate steroids, such as betamethasone, methylprednisolone, and triamcinolone, might be bigger in size than red blood cells.^[10]^ Thus, these particles may formulate emboli in arteries, arterioles, or arterial capillaries if injected into the radicular artery.^[14]^ The possibility of SCI by embolization can be considered even in this case because triamcinolone was used. However, asserting that this factor is the only cause of this case is difficult because the Adamkiewicz artery should have been occluded when considering the T7 to L2 level involvement, but the injection site was fairly far from the Adamkiewicz artery. Adamkiewicz artery, also known as the great anterior medullary artery, is a major artery responsible for arterial blood supply to the spinal cord from the T8 level to the conus medullaris,^[15]^ and most often a single vessel (87.4%) originating between the T8 and L1 levels (89%).^[16]^ Therefore, ESI-related SCI has been usually reported in the case of the upper-mid lumbar region but rarely occurs in the lower lumbar or sacral region.^[17]^ Furthermore, CEB-induced SCI has not been reported.

Another possible cause to consider is Tarlov cysts. Wang et al^[10]^ reported a similar case of a patient with tethered cord syndrome who experienced SCI following CEB.The authors indicate that a deeply inserted needle during the caudal injection may have pierced the dural sac, allowing the drugs to enter and cause SCI due to the presence of a tethered spinal cord, wherein the bottom of the dural sac is positioned too low. Additionally, a Tarlov cyst was observed at the S4 to S5 levels in our case, and the intracystic hemorrhage may have been caused by needle injury during CEB. Tarlov cyst is a cystic lesion located in the sacral region that communicate with the dural sac and are filled with cerebrospinal fluid. They are commonly observed in approximately 4.27% of cases, which are mostly asymptomatic.^[18]^ Hence, the Tarlov cyst can be considered to be punctured during the procedure in the present, and drug administration into the dural sac may have contributed to the occurrence of SCI, similar to the previous case.

Other notable case reports highlight the association between Tarlov cysts and SCI. Specifically, the occurrence of hemorrhage within a Tarlov cyst is rare and indicates a potential intracystic vascular malformation, such as an arteriovenous fistula. Yoshikawa et al^[19]^ reported a case of a sacral Tarlov cyst with intracystic hemorrhage and an accompanying arteriovenous fistula, which was treated with transcatheter arterial embolization. Additionally, Duja et al^[20]^ reported a case of a hemorrhagic Tarlov cyst associated with cerebral infarction, possibly due to cerebrospinal fat embolism following cyst rupture, although not directly related to SCI. This indicates a vascular malformation within the Tarlov cyst in the present case, and vascular administration of fat embolism or particulate steroids might occur after hemorrhagic rupture, which finally caused SCI. However, establishing an exact relationship remains a challeng because of inadequate additional angiography in the presented case.

Finally, vasospasm can be another potential cause of SCI. Vasospasm or vasoconstriction can be caused by various factors, including the use of drugs, such as cocaine and amphetamines, blood vessel endothelial lining damage, blood clot formation within vessel walls, and inflammatory reactions.^[21–24]^ Vasospasm is also often accompanied by hemorrhagic events, such as subarachnoid hemorrhage, and causes severe complications.^[22]^ Vasoconstrictors play a role in protecting the blood vessel lining by constricting the muscles within them in case of hemorrhage. However, the concentration of vasoconstrictors in the blood may increase or their ability to protect the blood vessel lining may be compromised in certain circumstances. In our case, hemorrhage from the cyst might induce autonomic blood vessel regulation, potentially causing radicular artery vasospasm.^[3]^ Notably, the onset of the sharp tingling pain and paralysis occurred right after CEB, indicating that a protective response triggered by hemorrhage in a Tarlov cyst may have induced immediate vasospasm and subsequently ischemia of the thoracolumbar spinal cord region.

## 4. Conclusion

CEB is a widely utilized and safe procedure, but the rare occurrence of severe complications should be acknowledged. In this particular case, the patient experienced the simultaneous onset of CES and SCI, suspected to be associated with a hemorrhagic injury of a sacral Tarlov cyst. Therefore, incorporating imaging techniques, such as MRI, before CEB procedures should be recommended to identify and assess the presence of Tarlov cysts, which may help mitigate potential risks and enhance patient safety.

## Author contributions

**Conceptualization:** Chung Reen Kim, Sunyoung Kim.

**Writing – original draft:** Sunyoung Joo.

**Writing – review & editing:** Chung Reen Kim, Sunyoung Kim.
